# DNA methylation patterns of candidate genes regulated by thymine DNA glycosylase in patients with *TP53* germline mutations

**DOI:** 10.1590/1414-431X20154026

**Published:** 2015-04-28

**Authors:** F.P. Fortes, H. Kuasne, F.A. Marchi, P.M. Miranda, S.R. Rogatto, M.I. Achatz

**Affiliations:** 1 CIPE, Laboratrio de Oncogentica Molecular, A.C. Camargo Cancer Center, São Paulo, SP, Brasil; 2 CIPE, Laboratrio NeoGene, A.C. Camargo Cancer Center, São Paulo, SP, Brasil; 3 Departamento de Urologia, Faculdade de Medicina, Universidade Estadual Paulista, Botucatu, SP, Brasil; 4 Programa Inter-Institucional em Bioinformtica, Instituto de Matemtica e Estatstica, Universidade So Paulo, So Paulo, SP, Brasil; 5 Departamento de Oncogentica, A.C. Camargo Cancer Center, So Paulo, SP, Brasil

**Keywords:** Li-Fraumeni syndrome, *TP53* gene, TDG, Methylation

## Abstract

Li-Fraumeni syndrome (LFS) is a rare, autosomal dominant, hereditary cancer predisposition disorder. In Brazil, the p.R337H *TP53* founder mutation causes the variant form of LFS, Li-Fraumeni-like syndrome. The occurrence of cancer and age of disease onset are known to vary, even in patients carrying the same mutation, and several mechanisms such as genetic and epigenetic alterations may be involved in this variability. However, the extent of involvement of such events has not been clarified. It is well established that p53 regulates several pathways, including the thymine DNA glycosylase (TDG) pathway, which regulates the DNA methylation of several genes. This study aimed to identify the DNA methylation pattern of genes potentially related to the TDG pathway (*CDKN2A*, *FOXA1*, *HOXD8*, *OCT4*, *SOX2*, and *SOX17*) in 30 patients with germline *TP53*mutations, 10 patients with wild-type *TP53*, and 10 healthy individuals. We also evaluated TDG expression in patients with adrenocortical tumors (ADR) with and without the p.R337H *TP53* mutation. Gene methylation patterns of peripheral blood DNA samples assessed by pyrosequencing revealed no significant differences between the three groups. However, increased *TDG* expression was observed by quantitative reverse transcription PCR in p.R337H carriers with ADR. Considering the rarity of this phenotype and the relevance of these findings, further studies using a larger sample set are necessary to confirm our results.

## Introduction

Li-Fraumeni syndrome (LFS, OMIM 151623) is an autosomal dominant disorder characterized by an inherited predisposition to cancer and the development of multiple primary tumors at an early age. The cancers most frequently associated with LFS are breast cancer, adrenocortical carcinoma (ADR), soft tissue sarcoma, osteosarcoma, and central nervous system tumors (1-4).

The main molecular mechanism underlying LFS is germline mutations in *TP53* (5), which predominantly occur in the central DNA-binding domain (6). In Southern Brazil, a variant form of LFS, Li-Fraumeni-like syndrome (LFL), occurs as a result of a founder mutation in exon 10 of *TP53*, replacing an arginine with histidine at codon 337 (p.R337H), which falls within the oligomerization domain (7). The p.R337H mutation alters the functional properties of the p53 protein at elevated intracellular pH values (above 7.0) and/or temperatures above 36.5C (7,8).

Recent reports have indicated that *TP53* mutations indirectly alter the levels of several transcripts (9), including the specialized base excision repair enzyme thymine-DNA glycosylase (TDG) (10). The primary role of TDG is to correct guanine:thymine and guanine:uracil DNA mismatches that result from the spontaneous deamination of 5-methyl cytosine and cytosine at CpG sites. These mutations can result in the loss of CpG dinucleotides, potentially affecting gene regulation (11,12). Lger et al. (13) demonstrated that the introduction of a P65A point mutation in *TDG*led to a significant loss of TDG/CREB-binding protein/retinoic acid receptor ternary complex stability, resulting in the deregulation of networks associated with DNA replication, recombination, and repair. TDG is also involved in the physiological control of promoter demethylation of several genes that are involved in embryogenesis and development (14-16), and it acts as a positive Wnt pathway regulator in patients with colorectal cancer (17).

Cells lacking TDG activity exhibit two major alterations: a decreased capacity for base excision repair, leading to increased sensitivity to mutagenic damage and the accumulation of mutations, and an impaired ability to maintain wild-type promoter region methylation patterns, resulting in inappropriate gene expression. Both TDG and ten-eleven-translocation (TET) protein mediate the demethylation and reactivation of micro (mi)RNAs that are critical for the mesenchymal-to-epithelial transition (18).

Alterations in the normal methylation patterns of TDG-regulated genes may be one mechanism underlying the occurrence of early age tumors in patients with germline *TP53* mutations. Indeed, the presence of high methylation levels in the promoter regions of certain genes has been considered to be a marker for several tumors (19-21). Epigenetic alterations, particularly DNA methylation, are a plausible molecular mechanism that may contribute to the diversity of tumors described in LFS/LFL patients.

p53 is known to alter TDG expression, which then modifies the methylation of genes related to embryogenesis and development. Our objective for the present study was therefore to evaluate the methylation patterns of six genes that are likely to be dependent on TDG activity, aiming to verify its relevance in patients carrying the p.R337H mutation. This group of genes produces transcripts that are related to pluripotency (*OCT4* and *SOX2*), a transcription factor involved in morphogenesis and a homeobox family member (*HOXD8*), a regulator of development (*SOX17*), a replicative senescence controller (*CDKN2A*), and a transcription factor related to embryonic development (*FOXA1*). We also used a retrotransposon sequence with constitutive, stable methylation (ALUyB8) as a DNA methylation control.

## Material and Methods

Fifty individuals recruited from the Oncogenetics Department of the A.C. Camargo Cancer Center (So Paulo, SP, Brazil) were selected for methylation analysis and divided into five groups: 1) 10 patient p.R337H carriers that had developed cancer, 2) 10 patient p.R337H carriers without cancer, 3) 10 individuals with cancer and carrying germline *TP53* mutations other than p.R337H, 4) 10 individuals with wild-type *TP53* and relatives who are carriers of germline *TP53* mutations, and 5) 10 healthy individuals with no personal or family history of cancer (Supplementary Table S1). All methylation assays were performed on DNA extracted from peripheral blood samples. Adrenocortical carcinomas from two patients with the p.R337H mutation and six patients without it were selected for gene expression analysis (Supplementary Table S2). The Oncogenetics Department of the A.C. Camargo Cancer Center followed up all patients. The Institutional Review Board approved this study (1669/12).

### DNA and RNA extraction

DNA was extracted from peripheral blood samples using a Gentra Puregene Blood kit (Qiagen, USA) according to the manufacturers instructions, quantified using a NanoDrop ND-1000 Spectrophotometer v.3.0.1 (Thermo Scientific, USA) and stored at 20C.

Total RNA was obtained from adrenocortical carcinomas using an RNeasy kit (Qiagen) according to the manufacturers recommendations. The quantity and quality of isolated RNAs were assessed using a NanoDrop ND-1000 Spectrophotometer v.3.0.1 (Thermo Scientific), and an Agilent 2100 Bioanalyzer (Agilent Technologies, USA) combined with an RNA 6000 NanoLabChip kit 2100 (Agilent Technologies), respectively. DNAs and RNAs were extracted from the samples at the A.C. Camargo Cancer Biobank (Brazil).

### Investigation of the germline *TP53* p.R337H mutation

Exon 10 of *TP53* was amplified using primer sequences 5-CAA CTT TTG TAA GAA CCA TC-3 and 5-GGA TGA GAA TGG AAT CCT AT-3 (22). Briefly, the amplification consisted of 35 cycles of denaturation at 94C, annealing at 57C, and extension at 68C. The PCR products were digested with 1U/L *Hha*I (Fermentas Inc., USA) for 16h at 37C, run on a 2 agarose gel (1 Tris-borate-EDTA buffer), and the following digestion patterns observed: 168 and 92bp bands indicating normal homozygous cells, 260, 168, and 92bp bands indicating p.R337H heterozygous cells, and a single 260bp band indicating p.R337H homozygous cells (23).

In addition to the p.R337H mutation, we also examined *TP53* exons 2-11, including the flanking intronic regions containing splice sites using protocols from the International Agency for Research on Cancer (http://p53.iarc.fr/Download/TP53DirectSequencingIARC.pdf). Sanger sequencing was conducted as described by Coulson (24). PCR amplification used a GeneAmp PCR System 9700 (Applied Biosystems, USA) and sequencing was performed using an ABI Prism Model 3130xl (Applied Biosystems) automatic sequencer. The resulting sequences were comparatively analyzed using a reference sequence (RefSeq NM000546.4) and the CLC Main Workbench 5.0.2 software (Denmark). This analysis included all exons and exon-intron junctions.

### Pyrosequencing investigation of methylated CpG islands

The presence of methylated CpG islands was examined in six genes: *FOXA1*, *OCT4*, *SOX17*, *CDKN2A*, *HOXD8*, and *SOX2*. A total of 500ng of DNA from each sample was treated with bisulfite using an EZ DNA Methylation Kit-Gold kit (Zymo Research, USA). *FOXA1*, *OCT4*, and *SOX17* amplification primers are described in Table 1. Standardized Qiagen tests were used for *CDKN2A*, *HOXD8*, and *SOX2* (Table 1).

**Table:**
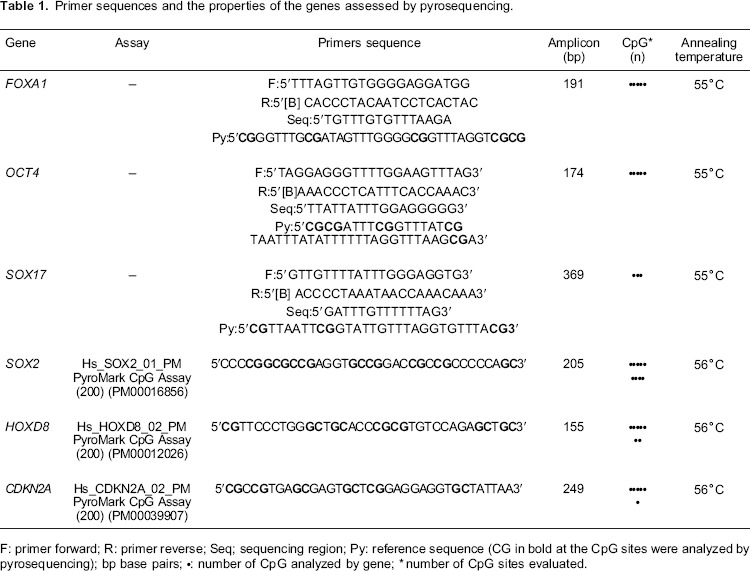

PCR amplifications were performed in 50-L volumes containing 1-L converted DNA (25ng), 10 buffer, 15mM MgCl_2_, 10mM of each dinucleotide, 10mM of each primer (one labeled with biotin at the 5 end), and 1U HotStartTaq DNA polymerase (Qiagen). Pyrosequencing reactions were performed using Pyromark Gold Q96 reagents (Qiagen) according to the manufacturers recommendations. Significant differences between groups were determined using the Kruskal Wallis test.

### cDNA synthesis and quantitative reverse transcription PCR analysis

To assess changes in *TDG* expression, RNA samples from eight adrenocortical carcinomas were used for cDNA synthesis as previously described (25). Quantitative reverse transcription (qRT)-PCR was performed using Power SYBR Green fluorescent dye (Applied Biosystems) in an ABI Prism 7500 Sequence Detection System. All sample values were normalized by dividing the values obtained for the gene of interest (*TDG*) with those for the reference genes (*HPRT* and *GAPDH*). The primers for transcript amplification were designed using the Primer Blast program (http://www.ncbi.nlm.nih.gov/tools/primer-blast/) (Supplementary Table S3). Target gene quantification was performed using Ct values and the 2^Ct^ formula (26). Expression values were compared with a control sample that consisted of a commercial RNA pool from normal adrenal tissue (Clontech, USA).

## Results

We identified 20 *TP53* p.R337H carrier patients, 10 patients with other *TP53* mutations, and 20 patients with wild-type *TP53*. Clinical and biological characteristics of the LFS/LFL patients, as well as the sequencing results for each case, are described in Supplementary Table S1.

**Figure 1:**
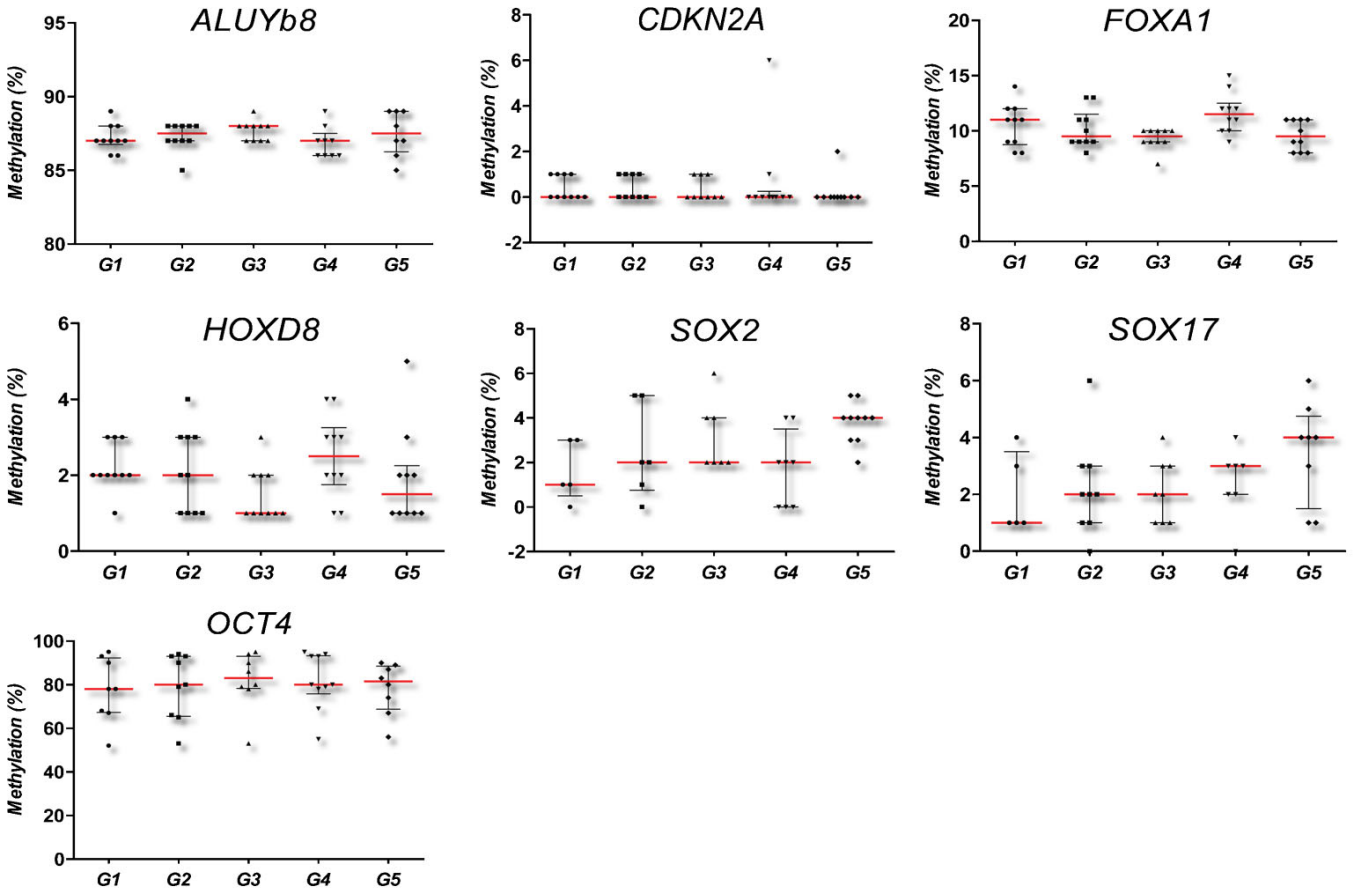
Dot plots representing the methylation levels of *CDKN2A*, *FOXA1*, *HOXD8*, *SOX2*, *SOX17* and *OCT4* candidate genes, as well as of the ALUYb8 control region, in groups G1 through G5. Groups: G1: 10 patient p.R337H carriers that had developed cancer G2: 10 patient p.R337H carriers without cancer G3: 10 individuals with cancer and carrying germline *TP53* mutations other than p.R337H G4: 10 individuals with wild-type *TP53* and relatives who are carriers of germline *TP53* mutations G5: 10 healthy individuals with no personal or family history of cancer. There were no significant differences (P0.05, Kruskal-Wallis test).

The methylation patterns of *CDKN2A*, *FOXA1*, *HOXD8*, *OCT4*, *SOX2*, and *SOX17* were evaluated by pyrosequencing. As shown in Figure 1, no significant differences were observed when all tested patient groups were evaluated. The methylation levels of *CDKN2A*, *SOX2*, *SOX17*, and *HOXD8* were below 5 in all five groups. Additionally, *FOXA1* showed methylation levels below 15 in all groups. *OCT4* and ALUyb8 methylation levels were approximately 80.

In the adrenocortical carcinoma samples, two of the eight tumors possessed the p.R337H mutation (Supplementary Table S2). RT-qPCR also revealed higher *TDG* expression in both p.R337H-positive cases however, this finding could not be tested for statistical significance because of the small sample size.

## Discussion

LFS patients have a 90 risk of developing cancer during their lifetime (27). According to Chompret criteria, germline mutations in *TP53* are found in 70 of LFS cases (28) and in 29 of LFL families (29). The *TP53* p.R337H founder mutation was reported to be associated with Brazilian families with LFL in 2007 (2), and has an estimated population frequency of 0.3 in Southern and Southeastern regions of Brazil, where the incidence of adrenocortical carcinoma is 10- to 15-fold greater than in other countries (30,31).

Recently, da Costa et al. (10) reported that *TDG* expression is directly regulated by wild-type p53 protein, suggesting that the loss of p53 function may affect TDG-mediated processes. A limited number of studies have assessed *TDG* expression levels in tumors. Nettersheim et al. (32) reported high levels of *TDG* and *TET* transcripts in germ cell-derived tumors, while Peng et al. (33) reported that *TDG* hypermethylation and the consequent reduction of transcript expression led to an impairment of repair in multiple myeloma cell lines. Similarly, Yatsuoka et al. (34) observed decreased *TDG* expression in 21 pancreatic cancer cell lines. Interestingly, *TDG* expression levels appear to be epigenetically regulated by DNA methyltransferases, especially DNMT3L (35), and the miRNA-29 family (36).

In addition to its involvement in DNA damage repair, TDG has been shown to be involved in epigenetic regulation, protecting CpG islands from hypermethylation through interactions with DNA methyltransferases and histone acetyltransferases. Moreover, TDG glycosylase activity plays an active role in 5-methylcytosine removal and thus leads to gene activation through demethylation (10,37,38). TDG is also very active during development (15), and epigenetically regulates several genes associated with development and cell determination such as the homeobox family genes and other transcription factors (15).

The present study evaluated the methylation patterns of *CDKN2A*, *FOXA1*, *HOXD8*, *OCT4*, *SOX2*, and *SOX17* in peripheral blood samples from patients with germline *TP53* mutations and healthy individuals. The six genes selected are related to development and embryogenesis and are potentially regulated by TDG.

Methylation profiles may differ in various tissues within a single individual (39). The assessment of methylation status in both tumor and peripheral blood samples therefore has the potential to reveal differences that could help us better understand the tumor variability and penetrance observed in LFS *TP53* germline mutation carriers. Methylation pattern analysis using peripheral blood samples is also an effective, non-invasive alternative to investigating the tumor spectrum variability within the syndrome. Our initial hypothesis was that epigenetic alterations would be observed in blood samples from LFS/LFL patients or that altered methylation patterns could indicate indirect alterations to TDG expression.

LFS/LFL patients display a variety of tumor types over a wide age spectrum, and it has been observed that even if patients carry the same mutation, they do not always exhibit the same phenotype (40). Alterations to the methylation patterns of genes potentially regulated by TDG could act as risk modifiers, and could explain the differences in the ages of tumor onset and tumor subtypes described in this syndrome however, we were unable to confirm the differences in the methylation patterns of the tested genes and samples (Figure 1). Nevertheless, our LFS/LFL patient cohort is one of the largest described with germline *TP53*mutations, even though we had a restricted number of patients who fulfilled the inclusion criteria. None of the genes evaluated in LFS/LFL patients showed hypermethylation compared with controls, so they cannot be used as markers for the assessment of LFS/LFL phenotypes. The use of more robust platforms (e.g., large scale analysis) or next-generation sequencing to assess epigenetic alterations is likely to be more effective in finding TDG-regulated genes or other markers to evaluate such phenotypic differences. It is also worth noting that methylation is labile and thus may be influenced by several factors such as life habits and age. Although increased *TDG* expression was observed in two adrenocortical carcinomas from patients who were positive for the p.R337H mutation, it was not possible to infer the relationship between the p.R337H mutation and *TDG* levels because of the small number of cases. A larger cohort of patients with matched controls is therefore needed to better assess TDG as a clinical marker for tumor occurrence in LFS families however, this disease is a rare syndrome and the recruitment of a large number of patients remains a challenge.

## Supplementary Material

Click here to view[pdf].
